# Fecal Impaction in the Rectum and Rectosigmoid Colon Secondary to Sunflower Seed Ingestion

**DOI:** 10.1155/2021/4826867

**Published:** 2021-10-05

**Authors:** Alexander Lyons, Jamie Lee, Kristen Cares

**Affiliations:** ^1^Children's Hospital of Michigan, Detroit, MI, USA; ^2^Division of Pediatric Gastroenterology, Children's Hospital of Michigan, Detroit, MI, USA

## Abstract

A 35-month-old male who had eaten a bag of sunflower seeds initially presented to the emergency department (ED) with visible seeds in the anus and was discharged home with a stool softener after manual disimpaction. He then returned to the hospital 2 days later, and abdominal radiographs confirmed significant fecal material within the rectum and rectosigmoid colon. After failed oral and rectal laxative therapy attempts, subsequent disimpaction under anesthesia revealed an undigested sunflower seed bezoar in the rectum extending to the distal segment of his sigmoid colon. This case highlights the dangers and possible complications of seed ingestion even in small quantities in children along with the pathophysiology of impaction. This is one of the youngest cases reported in the United States involving the rectum and rectosigmoid colon with a sunflower bezoar.

## 1. Introduction

Fecal impaction, an accumulation of hard stool in the anorectum or distal colon, may occur due to a variety of primary causes, with the most common being a diet consisting of inadequate intake of fiber and fluids [1, 2]. Although seeds are a source of fiber, there is a crucial difference between soluble and insoluble fiber. Soluble fiber absorbs water when exposed to gastrointestinal fluids, forming a gel-like substance that is subsequently digested by bacteria in the large intestine, ultimately releasing gas. However, seeds are a source of insoluble fiber; therefore, they pass through the intestinal tract without being digested, which can lead to a bezoar [3]. The different types of bezoars include phytobezoars (fruit and vegetable fibers), trichobezoars (hair), lactobezoars (milk concretions), and pharmacobezoars (medications) [4]. In a majority of children, fecal impaction of seeds is found in the rectum followed by the ileum [1, 5].

## 2. Case Report

**Figure 1:**
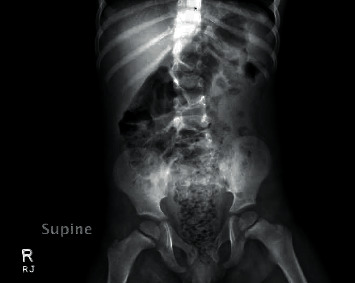
Abdominal X-ray, supine view. There is a nonobstructive gas pattern with moderate stool burden, primarily in the rectosigmoid colon and to a lesser extent in the ascending colon.

A previously healthy 35-month-old male (16.8 kg) initially presented to the emergency department (ED) with complaints of abdominal pain and difficulty passing stool for 2 days. Two days prior to the ED visit, the mother noted that the patient had consumed an entire bag of sunflower seeds. Following this, he developed constipation with straining during defecation, and sunflower seeds were seen protruding from the rectum, prompting his mother to bring him to the ED. In the ED, an abdominal X-ray revealed a nonobstructive bowel gas pattern with a moderate amount of stool seen in the colon and rectum (Figure 1). During an attempt to administer an enema, sunflower seeds were protruding from the rectum; therefore, several seeds were manually removed. He subsequently passed a small amount of stool and was then discharged home on a stool softener.

**Figure 2:**
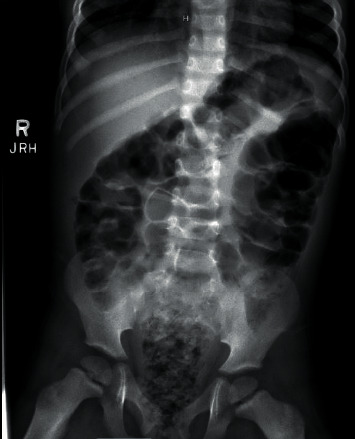
Abdominal X-ray, supine view. Following 2 days on stool softener and representation to the ED, there is mild gaseous distention of the visualized large colon with a moderate degree of fecal material within the rectum and rectosigmoid colon, not subsequently moved by mineral oil enema.

Two days following his discharge, the patient returned to the ED with persistent constipation, abdominal pain with straining, vomiting, as well as decreased appetite. His mother reported nonbloody liquid stools. An abdominal X-ray revealed gaseous distention of the colon with a moderate degree of fecal material in the rectum and rectosigmoid colon (Figure 2). A mineral oil enema was administered; however, it failed to produce any stool. The patient was admitted for an exam under anesthesia and manual disimpaction.

**Figure 3:**
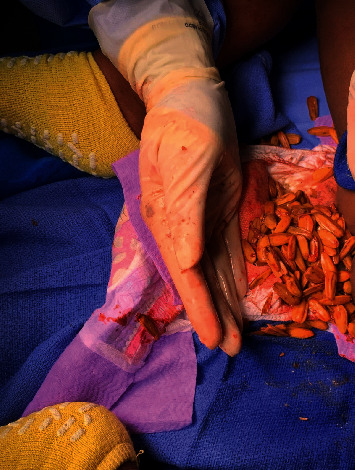
Sunflower seeds removed by manual disimpaction from the rectum and rectosigmoid colon. There is minimal fecal material with some specks of blood secondary to trauma. Photo was obtained with signed and informed consent.

Upon the digital rectal exam, a large number of sunflower seeds were palpable in the rectal vault with significant distention. Whole sunflower seeds were then evacuated from the rectum (Figure 3). There was minimal fecal material in the rectum. The sharp edges of the sunflower seeds also created mucosal trauma from within the rectum. All of the sunflower seeds within reach were removed. The patient was later discharged from the hospital on daily MiraLAX and Calmoseptine cream for any possible anorectal pain. He was subsequently lost to follow-up.

## 3. Discussion

Seeds can produce phytobezoars, which are accumulations of indigestible vegetables or fruit seeds in the intestinal tract. Unlike dietary fiber bezoars contained in fruits and vegetables, which more often accumulate in the stomach, seed bezoars are able to pass the pylorus and subsequently the ileocecal valve and accumulate in the colon and rectum due to their small size. Seed bezoars typically form an impaction in the rectum due to the decreased water absorption [4–6].

A systematic review of cases of gastrointestinal bezoars spanning from 1980 to 2018 revealed that the most common ethnicity groups affected were from the Eastern Mediterranean and Middle Eastern regions likely due to diets containing more fruits and vegetables, followed by Western Europe and the Americas. Affected children were predominately male (65%) with ages ranging from 2 to 16 years old with a median age of 10. Common locations of bezoar impaction included the rectum, which would explain the most common symptoms of abdominal and rectal pain. Other symptoms reported include tenesmus and loose stools. There were few case reports in children that presented with mildly elevated temperature and leukocytosis mimicking acute appendicitis, one of which involved a 3-year-old child that presented with fever, leukocytosis, and signs of colitis [7]. Of the different seed types found in bezoars, watermelon seeds comprised the majority (54%) followed by sunflower seeds (21%) [6]. Among reported cases, this is the second reported case in the United States with rectal and rectosigmoid colon involvement due to sunflower seeds.

Based on the 2014 recommendations from ESPGHAN and NASPGHAN, diagnosis of fecal impaction should be based upon the clinical history and digital rectal examination. Plain abdominal radiography may be used in children where fecal impaction is suspected and physical examination is unreliable or impossible [1]. The patient in our case met the diagnostic criteria for fecal impaction based on the physical examination and history. This highlights the importance of obtaining a sufficient dietary history and physical exam prior to getting abdominal X-rays. In instances where a bezoar causes obstruction in the small bowel, complications such as pancreatitis and rarely bowel perforation may occur and require computerized tomography for diagnosis and referral to surgery [6, 8, 9].

In patients with rectal seed impaction, manual disimpaction under general anesthesia has been the preferred treatment to minimize patient discomfort [6, 8, 9]. Initial conservative measures of intervention failed in our patient, which is a common occurrence in patients with fecal impaction, as only 6% of seed bezoars are typically removed with Fleet enemas and stool softeners [6].

## 4. Conclusion

Rectal bezoars should be diagnosed using a dietary history and physical exam. Given that the initial treatment in our patient was manual disimpaction without anesthesia, it partially removed the seed impaction. However, it could not remove any further impaction secondary to pain in the child. Understanding the pathophysiology of rectal bezoars and the success of manual disimpaction under general anesthesia will prove beneficial, as it should be used as a first-line treatment in children with a history of seed ingestion or visualization of seeds on rectal exam as in our patient, minimizing treatment failure.

## References

[1] Manatakis D. K., Acheimastos V., Antonopoulou M. I., Balalis D., Korkolis D. P. (2019). Gastrointestinal seed bezoars: a systematic review of case reports and case series. *Cureus*.

[2] Tabbers M. M., DiLorenzo C., Berger M. Y. (2014). Evaluation and treatment of functional constipation in infants and children. *Journal of Pediatric Gastroenterology and Nutrition*.

[3] Axelrod C., Saps M. (2018). The role of fiber in the treatment of functional gastrointestinal disorders in children. *Nutrients*.

[4] Eng K., Kay M. (2012). Gastrointestinal bezoars: history and current treatment paradigms. *Gastroenterology and Hepatology*.

[5] Eitan A., Katz I. M., Sweed Y., Bickel A. (2007). Fecal impaction in children: report of 53 cases of rectal seed bezoars. *Journal of Pediatric Surgery*.

[6] Iwamuro M., Okada H., Matsueda K. (2015). Review of the diagnosis and management of gastrointestinal bezoars. *World Journal of Gastrointestinal Endoscopy*.

[7] Efrati Y., Freud E., Serour F., Klin B. (1997). Phytobezoar-induced ileal and colonic obstruction in childhood. *Journal of Pediatric Gastroenterology and Nutrition*.

[8] Chen Y.-C., Liu C.-H., Hsu H.-H. (2015). Imaging differentiation of phytobezoar and small-bowel faeces: CT characteristics with quantitative analysis in patients with small-bowel obstruction. *European Radiology*.

[9] Lane R. D., Schunk J. E. (2010). Sunflower rectal bezoar presenting with an acute abdomen in a 3-year-old child. *Pediatric Emergency Care*.

